# Effect of Embryonic Alcohol Exposure on Craniofacial and Skin Melanocyte Development: Insights from Zebrafish (*Danio rerio*)

**DOI:** 10.3390/toxics10090544

**Published:** 2022-09-18

**Authors:** Parnia Azimian Zavareh, Praneeth Silva, Nuwanthika Gimhani, Devi Atukorallaya

**Affiliations:** Department of Oral Biology, Dr. Gerald Niznick College of Dentistry, Rady Faculty of Health Sciences, University of Manitoba, Winnipeg, MB R3E 0W2, Canada

**Keywords:** zebrafish, teeth, ethmoid bone, melanocytes, neural crest cells, fetal alcohol spectrum disorder

## Abstract

Alcohol is a common addictive substance and prenatal alcohol exposure could cause fetal alcohol spectrum disorder (FASD) and can lead to various birth defects. The small teleost zebrafish (*Danio rerio*) has been identified as a fine animal model in developmental biology and toxicological research. Zebrafish models are widely used to study the harmful effects of alcohol and limited studies are available on the craniofacial and skin malformations associated with FASD. The present study attempts to investigate the effect of alcohol on early zebrafish embryonic development. The effects of prenatal alcohol exposure on neural crest cell-derived organ formation, including pharyngeal dentition, palatal bones and skin melanocytes were analysed. Whole-mount cartilage and bone staining and imaging techniques were applied to determine the effects of alcohol on the above-mentioned structures. The tooth size and shape were affected by alcohol exposure, but the number of teeth in the pharyngeal dentition was not affected. Only first-generation teeth showed size differences. The alcohol-exposed ethmoid bone, which is homologous to the human hard palate, was smaller and less dense in cell arrangement compared with the control medial ethmoid bone. The skin pigmentation defects included reduced melanocyte density, melanin contraction, smaller melanocyte surface area and aberrations in melanosome dispersion, revealing that alcohol significantly influenced and downregulated each and every step of the melanocyte developmental process. This descriptive study summarises the effects of alcohol on the development of neural crest cell-derived structures and highlights the importance of zebrafish in studying the phenotypic characteristics of fetal alcohol spectrum disorder.

## 1. Introduction

Alcohol is a commonly used and popular psychoactive drug. Multiple studies have confirmed that prenatal alcohol exposure (PAE) is likely one of the leading causes of birth defects including craniofacial, cardiac, ocular and neural phenotypic defects, and cognitive behavioural impairments [1,2,3,4,5,6]. Consuming alcohol during pregnancy is a serious health problem because it has negative effects on the developing embryo. Presently, alcohol-induced birth defects are collectively termed as FASD. The global prevalence of FASD is 0.7%, with a higher prevalence of 2–5% in Europe and North America, highlighting the need for increased diagnosis and treatment [7]. Embryonic exposure to alcohol has been consistently associated with characteristic gross defects in developing embryos, with varying degrees of physical abnormalities and developmental delays, alongside increased morbidity and mortality. Zebrafish embryos provide more advantages than *Xenopus*, murine and other mammalian models in terms of studying prenatal alcohol exposure [8,9]. Recent studies have highlighted the use of zebrafish embryos as a model for embryonic alcohol exposure [10,11,12]. Moreover, this small teleost fish model is being used to identify and characterise the gene–alcohol interactions that underlie FASD and has also provided important information for understanding the pathological basis of FASD-associated phenotypic body defects [2].

The severity of FASD mainly depends on the time, duration, frequency and quantity of alcohol exposure as well as genetic make-up [13,14,15]. Alcohol can exert numerous teratogenic effects. It can affect gametogenesis and preimplantation and gastrulation of embryogenesis and can also cause epigenetic modifications that lead to the manifestation of FASD [16,17]. These effects occur through numerous mechanisms. Alcohol competitively affects the retinoic acid pathway [18,19,20], Sonic hedgehog pathway [21,22,23] and insulin signalling pathways and causes varying degrees of developmental malformations including craniofacial defects [24]. Cellular defects are initiated by inhibition of some enzymes that are produced in the Golgi apparatus and impair cellular interactions. In addition, the formation of reactive free radicals such as the superoxide anion radical (·O_2_), hydrogen peroxide (H_2_O_2_) and the hydroxyl radical (·OH) within the cell during alcohol metabolism inhibit cell differentiation, disturb cell–cell interactions and impair cellular metabolism [25,26].

Neural crest cells form in the dorsal-most part of the nascent embryonic central nervous system, from which they detach and then migrate throughout the embryo to give rise to a diverse array of cell types. Neural crest cells make up many of the morphological and physiological traits that characterise the vertebrate clade [27,28]. Research has shown that alcohol adversely affects multiple events of neural crest cell development [29,30,31]. Moreover, alcohol exposure displays abnormal neural crest cell migration patterns such as loss of left–right symmetry and increased apoptosis [32]. The lower endogenous superoxide dismutase (SOD) activity in neural crest cells may enhance their sensitivity to the stress of reactive oxygen intermediates [33,34]. Oxidative stress and free radicals together contribute to the apoptosis of alcohol-exposed neural crest cells [25,26,35]. The development of cranial neural crest populations is most commonly affected by alcohol, with reductions in their derived facial bone and cartilage, cranial nerves, tooth structure and cardiac outflow tract [29,36]. However, there is no report on the effect of alcohol on neural crest cell-induced skin pigmentation [36].

In all vertebrates, teeth develop from the reciprocal interactions between the epithelium and neural crest cell-derived mesenchymal cells [37]. A zebrafish is a polyphyodont, meaning there are multiple tooth replacement cycles throughout its life. The tooth structure is homologous to human teeth. Zebrafish teeth are located in the pharyngeal region and on the fifth ceratobranchial (cb5) bone. This bone carries three rows of teeth, the ventral (V), mediodorsal (MD) and dorsal (D) rows, which present five ventral teeth (1 V–5 V), four mediodorsal teeth (1 MD–4 MD) and two dorsal teeth (1 D–2 D) in adult fish [38], respectively. In zebrafish, around 48 h post-fertilisation (hpf), the epithelium overlying the cb5 bone starts thickening [39]. The thickened epithelium gradually invaginates into the underlying mesenchyme to form an asymmetrical bell-shaped enamel organ [39]. Enameloid and dentin are formed with the cytodifferentiation of the cells in the enamel organ. Once fully formed, the teeth attach to the pharyngeal bone and erupt into the pharyngeal cavity. The first erupting tooth of zebrafish is 4V1 at 4 days post-fertilisation (dpf). There are a few differences between zebrafish and mammalian tooth development. For example, the crown and root of a zebrafish tooth is not distinguishable, the teeth are covered by a layer of hyper-mineralised enameloid rather than enamel and there are no dentinal tubules in the underlying dentin [40].

During palate development, the frontonasal mesenchyme process and the mesenchyme of the maxillary processes are derived from migratory neural crest cells. Previous research has suggested that the human primary palate is homologous to the medial ethmoid region and the secondary palate is homologous to the lateral ethmoid region of zebrafish [41,42]. The V-shaped fusion seam of the frontonasal process and the bilateral maxillary processes of the human palate are analogous to the V-shaped junction found in the zebrafish ethmoid cartilage. During median ethmoid cartilage development in zebrafish, discrete pairs of cranial neural crest cells in the anterior lateral direction merge and converge towards the midline [43]. After that, this group of cells makes a sharp 180° turn at the anterior boundary while condensing further into one group, streaming into the caudal direction to fuse with the paired lateral ethmoid cartilage [43]. Once the median ethmoid cells reach the paired lateral ethmoid region, integration of these parts begins to complete the fusion of the ethmoid cartilage. During this integration, juxtaposed columnar-shaped cells in the lateral ethmoid and cuboid-shaped cells in the median ethmoid undergo a morphological transition [43]. 

Vertebrate melanocytes are another cell type which originates from neural crest cells [44]. Humans have only one pigment cell type, the melanocyte, which produces the pigment melanin that is secreted into the skin. In contrast, basal vertebrates such as zebrafish develop several chromatophore types [45]. Zebrafish melanocytes are found in both epidermal and dermal tissue. Dermal melanophores are dendritic and located in the basal layer known as the hypodermis while epidermal melanophores are round and smaller, distributed in the scales and fins [46]. In humans, epidermal melanocytes are common; they are not associated with rapid chromatophore colour changes but rather with pigmentation of hair and skin. Moreover, these melanocytes cooperate with 30–40 associated keratinocytes and form the epidermal melanin unit [47].

This study was designed to develop zebrafish as an experimental model to investigate the effects of embryonic alcohol exposure on neural crest cell-derived organ development. We hypothesize that early embryonic exposure of alcohol has an effect on the size and shape of tooth and ethmoid bone as well as the morphology and density of melanocytes. We chose three organ systems: teeth, the palate and skin pigmentation. In tooth development, we observed deformed cusp morphology in the younger stages of alcohol-treated samples compared with the control. Ethmoid cartilage showed a reduction in the mean height and width and a lack of staining intensity in the medial ethmoid region after alcohol exposure. We found that embryonic alcohol exposure affected several stages of melanocyte development by decreasing the melanocyte density, surface area and pigment coverage. Our results revealed that tooth, ethmoid cartilage and skin melanocyte development were adversely affected by embryonic exposure to alcohol. 

## 2. Materials and Methods

### 2.1. Zebrafish Strains and Maintenance

These experiments were conducted on male and female wild-type AB zebrafish (*Danio rerio*), which were purchased from Zebrafish Genetics and Disease Models Core Facility, Hospital for Sick Children (Toronto, ON, Canada). The fish were housed and their colony established according to the Institutional Animal Care and Use Committee protocols at the central animal care facility at the Bannatyne campus, University of Manitoba. Adult zebrafish were fed a diet of Gemma 300-supplemented live shrimp and maintained on a 14/10 day/night cycle. Embryos were obtained from natural spawning. The collected eggs were cleaned and reared according to standard conditions. Fish were raised, bred and embryo collection was carried out according to the guidelines of the Canadian Council of Animal Care (CCAC) and protocols were approved annually by the Animal Care Committee, University of Manitoba. 

### 2.2. Alcohol Treatment and Embryo Fixation

Ten hours post-fertilisation, embryos were treated with 1% alcohol (Cat. No. HC13001GL, Fisher Scientific, Hampton, NH, USA). Control samples were kept in fish water. This alcohol concentration has been used previously and calculated as the effective alcohol concentration in fish alcohol research [48,49]. Twelve hours post-fertilisation, the embryos were taken out of the alcohol and washed 3–4 times with the fish water. Embryos were raised at 28.5 °C, changing the water once every day. They were euthanised at different ages by 1% tricaine methanesulphonate (MS222) (Cat. No. 118000500; Acros Organics, Hampton, NH, USA) and fixed overnight in 4% paraformaldehyde (PFA), then stored in phosphate-buffered saline. For each treatment, 12 embryos were used, and three biological replicates were collected for each experiment.

### 2.3. Whole-Mount Double Staining

For the tooth analysis, acid-free double cartilage and bone staining was performed to check the structural changes associated with the pharyngeal dentition [50]. Briefly, the fish were stained overnight in Alcian blue (Cat. No. AC400460250 Acros Organics, Hampton, NH, USA) and Alizarin red (Cat. No. LC105908, LabChem, Zelienople, PA, USA), rinsed in water and bleached in 3% hydrogen peroxide (Cat. No. 181926, Fisher Scientific, Hampton, NH, USA) in 1% potassium hydroxide (Cat. No. 134060010; Fisher Chemicals, Hampton, NH, USA) for 20 min. All specimens were processed through an ascending series of glycerol in 1% potassium hydroxide and then transferred to the storage solution (100% glycerol) [51].

### 2.4. Tooth Measurements

For tooth analysis, 12 fish samples were examined at 15, 20 and 25 dpf. The lower pharyngeal jaw was removed from each sample using fine dissection needles under a Nikon-SMZ 10A dissecting microscope (Nikon, Japan). The pharyngeal jaws were positioned with the dorsal side facing up and the rostral side facing west. They were mounted in 100% glycerol with coverslip. The sample was examined under a Zeiss discovery V8 stereomicroscope (Zeiss, Germany) and mounted on a binocular stereo microscope with 3.2×, 4× and 8× total magnification used to observe the tooth height and width, cusp shape and patterning, respectively. ZEN 2011 software (blue edition, Zeiss) was used to calculate the above-mentioned measurements.

**Figure 1:**
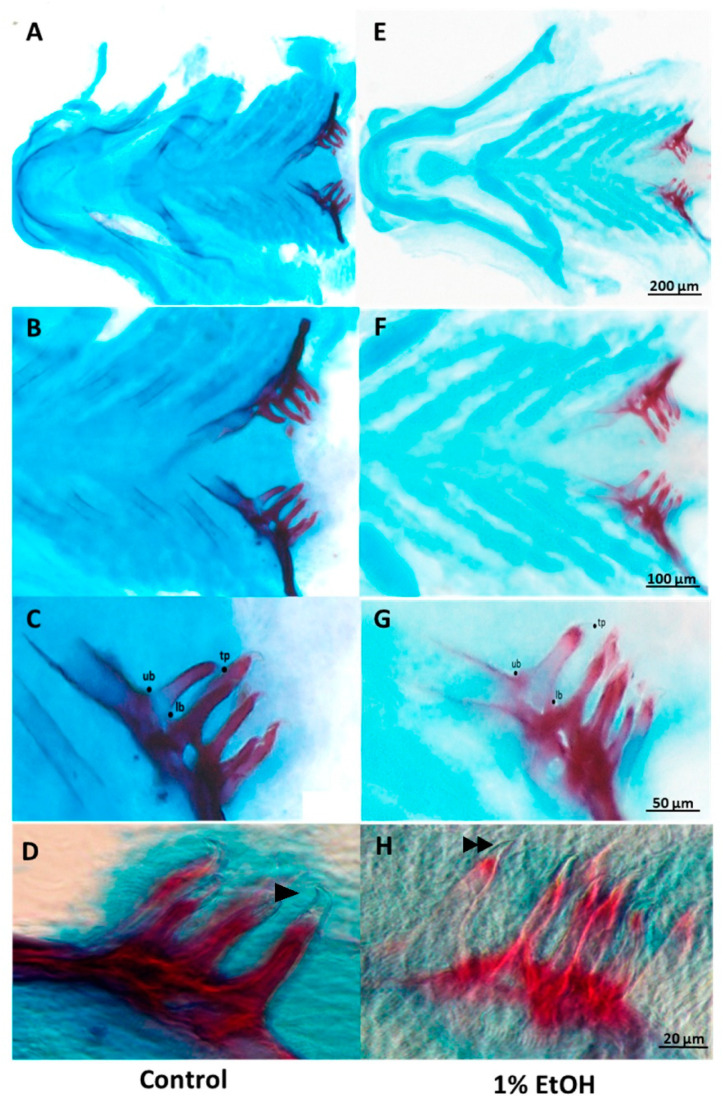
Acid-free double-stained tooth-bearing pharyngeal bones of zebrafish at 15 days post-fertilisation (dpf). (**A**–**D**) Control samples that show tooth-bearing lower pharyngeal bones with six teeth in each bone. These teeth were unicuspid and directly attached to the underlying bone. At this stage the teeth were fully mineralised. (**E**–**H**) Samples exposed to 1% alcohol at 10 h post-fertilisation showed small, malformed and hypo-mineralised teeth. (**D**,**H**) Enlarged view of the pharyngeal teeth with hooked cusp in control (arrowhead) and straight cusp in 1% alcohol-treated sample (double arrowhead) lb: lower base; tp: tip of the tooth; ub: upper base.

To measure the length and width of the pharyngeal tooth, the most rostral tooth in each age group was considered as a reference. In addition, three landmarks identified in all teeth were examined (Figure 1): the tip of the tooth (tp) and the uppermost (ub) and lowermost (lb) point of the base of the tooth relative to the downward curvature of the tooth towards the tip. Tooth length was the distance from tp to ub and tooth width was the distance from ub to lb [52].

### 2.5. Whole-Mount Cartilage Staining

Alcian blue staining was carried out as described previously with modifications [53]. The samples were stained for 3 h in 0.015% Alcian blue in 80% alcohol and 20% glacial acetic acid. Then, the samples were bleached in 0.8% potassium hydroxide, 0.9% hydrogen peroxide and 0.2% Triton X-100. After neutralisation in saturated sodium tetraborate, the tissue was softened in 0.2 mg/mL trypsin in 60% sodium tetraborate and 0.2% Triton X-100 and cleared in 18% glycerol, 0.8% potassium hydroxide and 0.2% Triton X-100.

### 2.6. Ethmoid Bone Measurements

The ethmoid bone was dissected from the skull using fine tungsten needles. The extracted bones were mounted on slides with glycerol and covered with a coverslip. The sample was examined under a Zeiss discovery V8 stereomicroscope. The height and width of the ethmoid palate were measured using the ZEN 2011 software (blue edition) for size and shape analysis. The height was measured as the maximum length from the rostral end of the ethmoid bone to the bifurcation of the lateral ethmoid bone. The width was measured at the maximum left to right width of the ethmoid bone perpendicular to height. Structural differences in the median ethmoid bone were visually checked by comparing the cell density of this area between control and alcohol treated samples.

### 2.7. Melanophore Density

Zebrafish were analysed from 4 to 10 dpf to identify the early number and developmental pattern of melanophores. Although PFA fixation can affect xanthophore and iridophore pigmentation, it does not affect melanin [54]. Embryos/larvae were mounted in 0.2% agar in embryo medium without a coverslip. A Region of Interest (ROI) was imaged in fixed fish by using a Zeiss discovery V8 stereomicroscope. This ROI encompassed the dorsal view of the head from midway up the eyes (excluding the eyes themselves) to the base of the head; pigmentation was measured from the dorsal pigment stripe only (Figure 7). Cells with an area ≥50% lying in the corresponding regions were included. Melanophores within the area of 91,656.991 µm^2^ of the ROI were used to manually count the number of melanocytes in the rostral portion of the dorsal pigment stripe. ZEN 2011 software (blue edition) was used for all measurements. The number of melanophores was plotted against the life stage and exact surface areas for each analysis.

### 2.8. Statistical Analysis

The data were subjected to one-way analysis of variance followed by Tukey’s pairwise comparison. The significance level was *p <* 0.05. Data were visualised in the form of box and interval plots. Minitab 17.3.1 software was used for the statistical analysis.

## 3. Results

### 3.1. Analysis of Tooth Morphology

**Figure 2:**
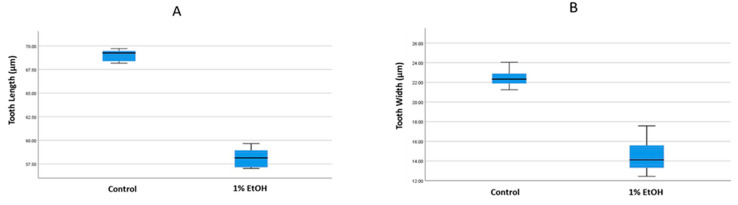
Comparing the tooth (**A**) length and (**B**) width in control and 1% alcohol-treated samples at 15 days post-fertilisation. In (**A**), the mean length is 69.71 µm for the control group and 58.23 µm for 1% alcohol-treated group. There was a significant difference (*p* < 0.05) between the groups. In (**B**), the mean width was 22.73 µm for control group and 14.22 µm for the 1% alcohol-treated group. There was a significant difference between the groups.

**Figure 3:**
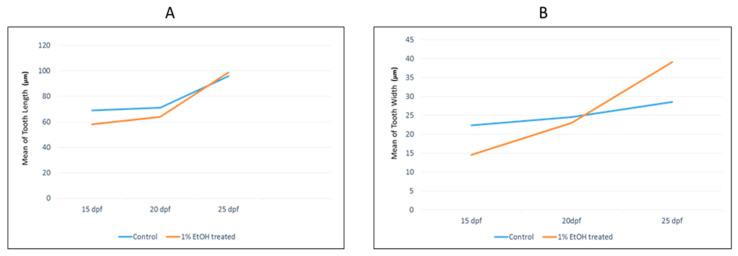
Comparing the mean tooth length in the control and 1% alcohol-treated samples at 15, 20 and 25 days post-fertilisation (dpf) (**A**). Comparing the mean tooth width in control and 1% alcohol-treated samples at 15, 20 and 25 dpf (**B**).

We stained mineralised teeth with Alizarin red in the acid-free double-staining method and analysed the associated structural changes induced by 1% alcohol exposure. The teeth of the alcohol-treated samples were deformed and showed a straight cusp morphology compared with the hook-like cusp in the control samples (Figure 1A). The cusp deformity differed significantly in the younger embryos and there were no differences in the older embryos. There was no significant change in the number of teeth in the alcohol-treated samples (*p* > 0.05). The height and width of the alcohol-treated teeth were significantly low compared to the control (Figure 2). The 15 dpf and 20 dpf samples (height 63.96 vs. 70.94 µm, width 22.98 vs. 24.55 µm) were significantly less than the control (*p* < 0.001 and *p* = 0.006, respectively). However, there was no significant difference in the height and width of the teeth between the 25 dpf control and alcohol-treated samples (*p* > 0.05) (Figure 3). These findings show that the adverse effects of alcohol were significant in first-generation teeth and then decreased through subsequent tooth replacement cycles. The dentition patterning was similar in the control and alcohol-treated samples. However, the first-generation ventral teeth of the 20 dpf alcohol-treated samples were exfoliated earlier than in the control samples.

### 3.2. Ethmoid Cartilage

**Figure 4:**
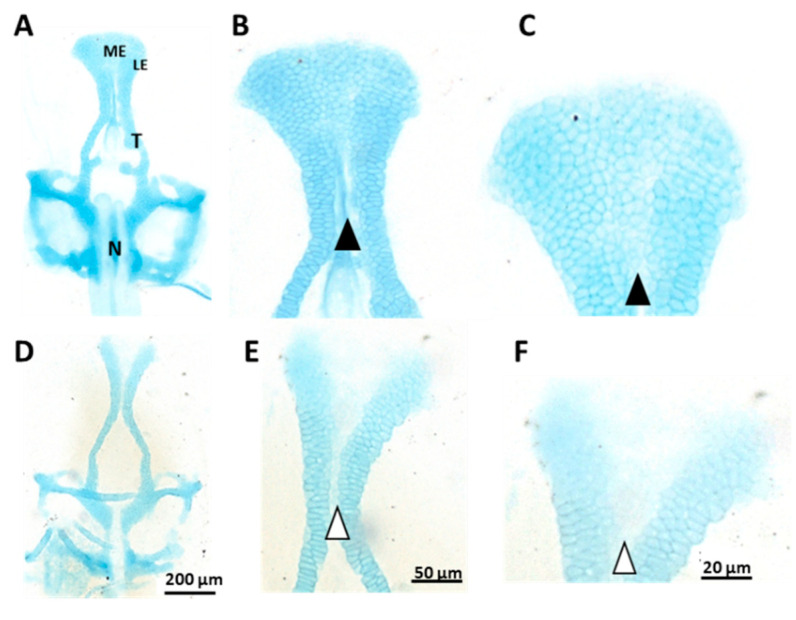
Alcian blue-stained (5 days post-fertilisation) (**A**–**C**) control and (**D**–**F**) 1% alcohol-treated zebrafish neurocranium (12 h treatment from 10 to 22 h post-fertilisation). Notice the lower cell density in the medial ethmoid region of (**E**,**F**) (white arrowheads) compared with (**B**,**C**) (black arrowheads). LE: lateral ethmoid cartilage; ME: medial ethmoid cartilage; N: notochord; T: trabeculae.

**Figure 5:**
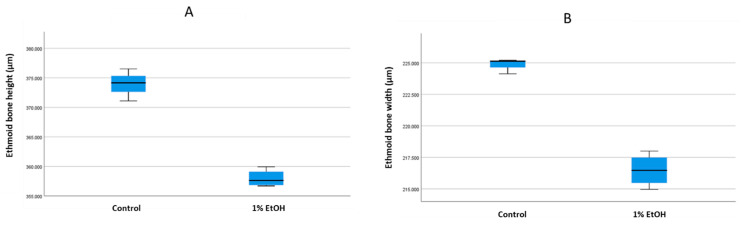
Comparing the ethmoid bone (**A**) height and (**B**) width in 10 days post-fertilisation (dpf) control and 1% alcohol-treated zebrafish samples. In (**A**), the mean length was 373.91 µm for control group and 357.97 µm for the 1% alcohol-treated group. There was a significant difference (*p* < 0.05) between the groups. In (**B**), the mean width was 224.80 µm for control group and 216.47 µm for 1% alcohol-treated group. There was a significant difference the groups.

We compared the size, shape and structure of the median ethmoid bone after 1% alcohol exposure. The overall shape of the ethmoid cartilage was not affected by alcohol exposure (Figure 4). However, there was less intense cartilage staining in the alcohol-treated samples compared with the control samples. In the medial ethmoid region there was a clear reduction in the cell density compared with the control (Figure 4E,F). We analysed the medial ethmoid region of the alcohol-treated samples at later ages (e.g., 10 and 15 dpf) and found no shape or size difference (results not shown). Next, we analysed the height and width of the ethmoid cartilage. We measured the mean height of the ethmoid cartilage as the maximum length from the tip of the ethmoid cartilage to the caudal end of the bifurcation of the bone. We measured the width as the widest length across the left and right cartilage plate. The mean height was 373.91 µm for the control samples and 357.97 µm for the alcohol-treated samples. The mean width was 224.80 µm for the control samples and 216.47 µm for the alcohol-treated samples (Figure 5).

### 3.3. Assessment of Melanocyte Density

**Figure 6:**
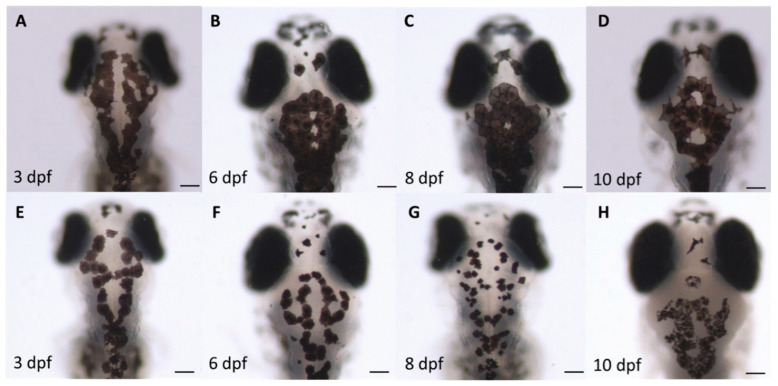
Comparison of the melanocyte density and morphology of the (**A**–**D**) control and (**E**–**H**) 1% alcohol-treated zebrafish embryos at 3, 6, 8 and 10 days post-fertilisation (dpf). The images illustrate the difference in melanocyte development in 1% alcohol-treated samples compared with control samples of each life stage, with defects in melanogenesis; melanin dispersion; and melanocyte surface area, arrangement and density. Scale bar: 100 µm.

**Figure 7:**
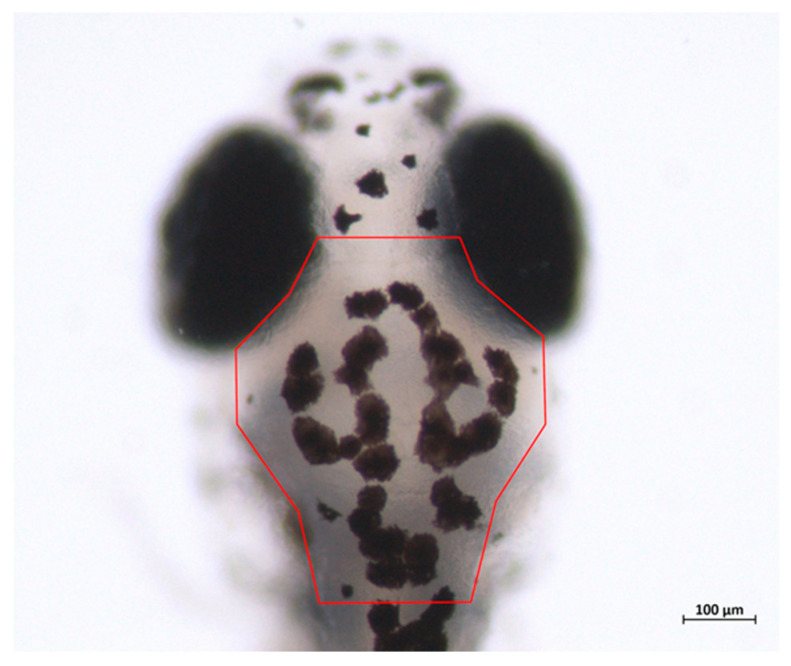
Region of interest (ROI) where the melanocyte density was compared between the control and 1% alcohol-treated zebrafish embryos at different developmental stages. (Red colour outline indicates the ROI). The ROI is defined as the area between the midline of the orbs and the base of the head, located most anterior part of the dorsal stripe.

There was a reduction in the number of melanocytes at each life stage of the alcohol-treated samples compared with the control samples (Figure 6). The embryos raised in alcohol had a lower mean pigmentation coverage of the region of interest (ROI), (Figure 7) compared with the control embryos. The melanocyte density pattern difference was more marked at 8 dpf (Figure 6C compared to the G). There were differences in the total surface area, size, arrangement and melanosome number of the melanocytes of the alcohol-treated samples compared with the control samples.

## 4. Discussion

In this study, we exposed zebrafish to 1% alcohol (171 mM) and investigated its effects on neural crest cell-derived organs. Neural crest cells form in the dorsal-most part of the nascent embryonic central nervous system. These cells then detach and migrate throughout the embryo to give rise to a diverse array of cell types that make up many of the morphological and physiological traits that characterise the vertebrate clade, including most of the craniofacial skeleton and peripheral sensory nervous system; striking patterns of pigmentation; components of the teeth, heart and endocrine system; and much more [55,56].

Alcohol is known to have a strong effect on craniofacial structures, particularly on tooth development [57,58]. Some animal studies have focussed on the effect of alcohol on developing dentition [59,60]. Researchers have reported that alcohol can cause cellular alterations in the inner enamel epithelium of the tooth germ during the bud stage and can influence the secretion of ameloblasts, which in turn can influence enamel formation [58,61]. Moreover, alcohol exposure in pregnant mini-pigs produces ultrastructural changes in secretory ameloblasts, retardation of cell differentiation within the tooth germ and calcification of the dentin matrix [62]. In this study zebrafish pharyngeal teeth showed an acute sensitivity to alcohol at different ages. Alcohol decreased the tooth length and width and altered mineralisation and the cusp shape, but only for first-generation teeth. In the older samples (>25 dpf), there were no differences between the alcohol-treated and control samples (Figure 3A,B). The tooth pattern was also the same in the control and alcohol-treated samples, whereas tooth exfoliation seemed to happen earlier in the alcohol-treated samples due to the hypo-mineralisation of the pharyngeal bone. 

This alcohol-induced craniofacial dysmorphogenesis was mainly the result of increased apoptosis, regenerative capacity and compensated facial primordial growth. Furthermore, after alcohol exposure, the zebrafish showed a malformed body cavity and fin displacement. In the ethmoid cartilage, the chondrocytes were well packed and stacked in the median and lateral ethmoid cartilage areas in the control sample (Figure 4B). Alcian-blue-staining intensity was similar between the median ethmoid and lateral ethmoid regions in the control samples. In contrast, the alcohol-treated samples presented a hypo-stained area in the median ethmoid region (Figure 4C,F). Because Alcian blue binds sulphated glycosaminoglycans (GAGs) or glycoproteins, a reduced staining intensity could result from less Alcian blue binding to glycoproteins in the cells of the median ethmoid region [63]. Moreover, studies have reported that alcohol can suppress the extent of neural crest cell migration from the prosencephalon, mesencephalon and rhombencephalon of the developing neural tube. Diminished cell migration could reduce the cell density and size in the area that develops from the corresponding neural crest cells, consistent with the significant reductions in the height (Figure 5A) and width (Figure 5B) of the ethmoid cartilage after alcohol treatment. 

According to the available literature, alcohol also affects other cranial bones. Among the most alcohol-sensitive structures are the eye, the otic capsule and the ethmoid plate. This alcohol-induced craniofacial dysmorphogenesis is mainly the result of increased cell apoptosis, regenerative capacity and compensated facial primordial growth [64]. Further, after alcohol exposure zebrafish show a malformed body cavity and fin displacement [64]. When exposed to alcohol, a zebrafish mutant with histone H4 transcription factor (*hinf*), polo-like kinase 1 (*plk1*), forkhead box 1 (*foxi1*) and methionyl-tRNA synthetase (*mars*) knockout shows a smaller ventral viscerocranium, microcephaly and microphthalmia [65]. Interestingly, there are severe craniofacial defects in the *plk1* mutant zebrafish, which exhibits a complete loss of the craniofacial skeleton, localised cell death and reduced axon projections [66]. Exposure to various other chemicals, including methotrexate, dexamethasone and chemicals present in smoke, can also cause defects in the ethmoid cartilage in zebrafish [67]. Exposure to 17β-oestradiol results in the cleft phenotype in the ethmoid cartilage in zebrafish [68].

Neural crest cell development involves cell-fate specification, proliferation, patterned cell migration, survival and differentiation. We observed melanogenesis defects after embryonic alcohol exposure due to the direct effects of alcohol on neural crest cell development. Melanocyte development is a complex process controlled by gene regulatory networks. Genetic alterations can lead to apparent morphological defects in melanocyte biology. A reduction in melanocyte density is caused by alterations in the specification of pigment cells from neural crest cells, proliferation of melanoblasts and the survival of melanocytes. Researchers have identified several candidate genes for melanogenesis. Mutations of the *white tail* (*wit*) and *colourless* (*cls*) genes in zebrafish generate a phenotype of melanophores with normal morphology and pigmentation but a reduced cell number or no cells [69].

Abnormal chromatophore patterning recorded on the head region of the dorsal region (Figure 6) involves errors in neural crest cell migration and localisation. Alterations in the melanophore surface area can lead to an abnormal chromatophore size and shape. These abnormalities are clearly influenced by mutants which are involved in chromatophore differentiation in terms of the expression of pigment or cell morphology. It could be greatly affected by the dominance of alcohol-induced mutations in the *obscure* (*obs*) and *union jack* (*uni*) genes [70]. As shown in Figure 6, the melanophores of the alcohol-treated samples were morphologically abnormal and deviated from the control samples. Either pattern was absent or formed incorrectly. Defects in the expression of the *choker* (*cho*) and *no tail* (*ntl*) genes were mainly responsible for this phenomenon [71,72].

## 5. Conclusions

Our findings demonstrated that embryonic alcohol exposure caused abnormal tooth formation, defects in palatal cartilage development and differentiation and patterning defects of melanocytes, suggesting the possible teratogenic effects of alcohol on neural crest cell-derived structures. The mechanisms of action of these phenotypic changes needs to be investigated further. These changes can be due to alcohol-induced changes, alcohol and gene interactions or it can be due to the activation or inhibition of the complex metabolic mechanisms within cells. The above phenotypes explained in this report will be further studied to identify the underlying genotypes. Specifically, gene–alcohol interactions associated with craniofacial and skin melanocytes will be investigated for specific cell-signalling pathway genes. Studies pertaining to the molecular mechanisms of gene–alcohol interactions will provide important information on identifying the aetiology of the visible phenotypic characters of FASD.

## Data Availability

Data is contained within the article.

## References

[1] DeRoo L.A., Wilcox A.J., Lie R.T., Romitti P.A., Pedersen D.A., Munger R.G., Moreno Uribe L.M., Wehby G.L. (2016). Maternal alcohol binge-drinking in the first trimester and the risk of orofacial clefts in offspring: A large population-based pooling study. Eur. J. Epidemiol..

[2] Lovely C.B., Fernandes Y., Eberhart J.K. (2016). Fishing for Fetal Alcohol Spectrum Disorders: Zebrafish as a Model for Ethanol Teratogenesis. Zebrafish.

[3] Cole G.J., Zhang C., Ojiaku P., Bell V., Devkota S., Mukhopadhyay S. (2012). Effects of ethanol exposure on nervous system development in zebrafish. Int. Rev. Cell Mol. Biol..

[4] Li X., Zhang L., Yin X., Gao Z., Zhang H., Liu X., Pan X., Li N., Yu Z. (2014). Retinoic acid remodels extracellular matrix (ECM) of cultured human fetal palate mesenchymal cells (hFPMCs) through down-regulation of TGF-beta/Smad signaling. Toxicol. Lett..

[5] Boschen K.E., Gong H., Murdaugh L.B., Parnell S.E. (2018). Knockdown of Mns1 increases susceptibility to craniofacial defects following gastrulation-stage alcohol exposure in mice. Alcohol. Clin. Exp. Res..

[6] Munger R.G., Romitti P.A., Daack-Hirsch S., Burns T.L., Murray J.C., Hanson J. (1996). Maternal alcohol use and risk of orofacial cleft birth defects. Teratology.

[7] Lange S., Probst C., Gmel G., Rehm J., Burd L., Popova S. (2017). Global Prevalence of Fetal Alcohol Spectrum Disorder Among Children and Youth: A Systematic Review and Meta-analysis. JAMA Pediatr..

[8] Cudd T.A. (2005). Animal model systems for the study of alcohol teratology. Exp. Biol. Med..

[9] Matsui J.I., Egana A.L., Sponholtz T.R., Adolph A.R., Dowling J.E. (2006). Effects of ethanol on photoreceptors and visual function in developing zebrafish. Investig. Ophthalmol. Vis. Sci..

[10] Arenzana F., Carvan III M.J., Aijon J., Sanchez-Gonzalez R., Arevalo R., Porteros A. (2006). Teratogenic effects of ethanol exposure on zebrafish visual system development. Neurotoxicol. Teratol..

[11] Bilotta J., Barnett J.A., Hancock L., Saszik S. (2004). Ethanol exposure alters zebrafish development: A novel model of fetal alcohol syndrome. Neurotoxicol. Teratol..

[12] Dlugos C.A., Rabin R.A. (2007). Ocular deficits associated with alcohol exposure during zebrafish development. J. Comp. Neurol..

[13] Hoyme H.E., May P.A., Kalberg W.O., Kodituwakku P., Gossage J.P., Trujillo P.M., Buckley D.G., Miller J.H., Aragon A.S., Khaole N. (2005). A practical clinical approach to diagnosis of fetal alcohol spectrum disorders: Clarification of the 1996 institute of medicine criteria. Pediatrics.

[14] Ouko L.A., Shantikumar K., Knezovich J., Haycock P., Schnugh D.J., Ramsay M. (2009). Effect of alcohol consumption on CpG methylation in the differentially methylated regions of H19 and IG-DMR in male gametes—Implications for fetal alcohol spectrum disorders. Alcohol. Clin. Exp. Res..

[15] Eaton B., Gangluff D., Mengel M. (2011). Fetal alcohol spectrum disorders: Flying under the radar. J. Ark. Med. Soc..

[16] Reik W., Dean W., Walter J. (2001). Epigenetic reprogramming in mammalian development. Science.

[17] Ramsay M. (2010). Genetic and epigenetic insights into fetal alcohol spectrum disorders. Genome Med..

[18] Deltour L., Ang H.L., Duester G. (1996). Ethanol inhibition of retinoic acid synthesis as a potential mechanism for fetal alcohol syndrome. FASEB J..

[19] Kot-Leibovich H., Fainsod A. (2009). Ethanol induces embryonic malformations by competing for retinaldehyde dehydrogenase activity during vertebrate gastrulation. Dis. Models Mech..

[20] Marrs J.A., Clendenon S.G., Ratcliffe D.R., Fielding S.M., Liu Q., Bosron W.F. (2010). Zebrafish fetal alcohol syndrome model: Effects of ethanol are rescued by retinoic acid supplement. Alcohol.

[21] Lipinski R.J., Godin E.A., O’leary-Moore S.K., Parnell S.E., Sulik K.K. Genesis of teratogen-induced holoprosencephaly in mice. American Journal of Medical Genetics Part C: Seminars in Medical Genetics.

[22] Li Y.-X., Yang H.-T., Zdanowicz M., Sicklick J.K., Qi Y., Camp T.J., Diehl A.M. (2007). Fetal alcohol exposure impairs Hedgehog cholesterol modification and signaling. Lab. Investig..

[23] De la Monte S.M., Wands J.R. (2010). Role of central nervous system insulin resistance in fetal alcohol spectrum disorders. J. Popul. Ther. Clin. Pharmacol. = J. De La Ther. Des Popul. Et De La Pharamcologie Clin..

[24] Haron M.H., Powe D., Khan I.A., Dasmahapatra A.K. (2012). Feasibility of medaka (Oryzias latipes) as an animal model to study fetal alcohol spectrum disorder. Advances in Molecular Toxicology.

[25] Cohen-Kerem R., Koren G. (2003). Antioxidants and fetal protection against ethanol teratogenicity: I. Review of the experimental data and implications to humans. Neurotoxicol. Teratol..

[26] Gemma S., Vichi S., Testai E. (2007). Metabolic and genetic factors contributing to alcohol induced effects and fetal alcohol syndrome. Neurosci. Biobehav. Rev..

[27] Eisen J.S., Weston J. (1993). Development of the neural crest in the zebrafish. Dev. Biol..

[28] Rocha M., Singh N., Ahsan K., Beiriger A., Prince V.E. (2020). Neural crest development: Insights from the zebrafish. Dev. Dyn..

[29] Smith S.M., Garic A., Flentke G.R., Berres M.E. (2014). Neural crest development in fetal alcohol syndrome. Birth Defects Res. Part C Embryo Today Rev..

[30] Rovasio R., Battiato N. (2002). Role of early migratory neural crest cells in developmental anomalies induced by ethanol. Int. J. Dev. Biol..

[31] Wang G., Bieberich E. (2010). Prenatal alcohol exposure triggers ceramide-induced apoptosis in neural crest-derived tissues concurrent with defective cranial development. Cell Death Dis..

[32] Garic A., Flentke G.R., Amberger E., Hernandez M., Smith S.M. (2011). CaMKII activation is a novel effector of alcohol’s neurotoxicity in neural crest stem/progenitor cells. J. Neurochem..

[33] Davis W., Crawford L., Cooper O., Farmer G., Thomas D., Freeman B. (1990). Ethanol induces the generation of reactive free radicals by neural crest cells in vitro. J. Craniofacial Genet. Dev. Biol..

[34] Chen S.y., Sulik K.K. (1996). Free radicals and ethanol-induced cytotoxicity in neural crest cells. Alcohol. Clin. Exp. Res..

[35] Gupta K.K., Gupta V.K., Shirasaka T. (2016). An Update on Fetal Alcohol Syndrome-Pathogenesis, Risks, and Treatment. Alcohol. Clin. Exp. Res..

[36] Czarnobaj J., Bagnall K.M., Bamforth J.S., Milos N.C. (2014). The different effects on cranial and trunk neural crest cell behaviour following exposure to a low concentration of alcohol in vitro. Arch. Oral Biol..

[37] Huysseune A., Sire J.-Y., Van der Heyden C. (1998). Initiation and development of cichlid and zebrafish first-generation teeth: An in vitro study. Biol. Jaarb. (Dodonaea).

[38] Huysseune A., Sire J.-Y. (2004). The role of epithelial remodelling in tooth eruption in larval zebrafish. Cell Tissue Res..

[39] Huysseune A., Thesleff I. (2004). Continuous tooth replacement: The possible involvement of epithelial stem cells. Bioessays.

[40] Zhang Y., Zhang Y., Zheng X., Xu R., He H., Duan X. (2016). Grading and quantification of dental fluorosis in zebrafish larva. Arch. Oral Biol..

[41] Mork L., Crump G. (2015). Zebrafish Craniofacial Development: A Window into Early Patterning. Curr. Top. Dev. Biol..

[42] Atukorala A.D.S., Ratnayake R.K. (2020). Cellular and molecular mechanisms in the development of a cleft lip and/or cleft palate; insights from zebrafish (Danio rerio). Anat. Rec..

[43] Schilling T.F., Kimmel C.B. (1997). Musculoskeletal patterning in the pharyngeal segments of the zebrafish embryo. Development.

[44] Brown G., Wellings S. (1970). Electron microscopy of the skin of the teleost, Hippoglossoides elassodon. Z. Für Zellforsch. Und Mikrosk. Anat..

[45] Fujii R. (1993). Cytophysiology of fish chromatophores. International Review of Cytology.

[46] Aspengren S., Hedberg D., Wallin M. (2006). Studies of pigment transfer between Xenopus laevis melanophores and fibroblasts in vitro and in vivo 1. Pigment Cell Res..

[47] Thody A., Shuster S. (1989). Melanophores, melanocytes and melanin: Endocrinology and pharmacology. Pharmacology of the Skin I.

[48] Dlugos C.A., Rabin R.A. (2003). Ethanol effects on three strains of zebrafish: Model system for genetic investigations. Pharmacol. Biochem. Behav..

[49] Lockwood B., Bjerke S., Kobayashi K., Guo S. (2004). Acute effects of alcohol on larval zebrafish: A genetic system for large-scale screening. Pharmacol. Biochem. Behav..

[50] Walker M., Kimmel C. (2007). A two-color acid-free cartilage and bone stain for zebrafish larvae. Biotech. Histochem..

[51] Aliesfehani T. (2015). Modified double skeletal staining protocols with Alizarinred and Alcian blue in laboratory animals. Ann. Mil. Health Sci. Res. • Vol.

[52] Yu J.C., Fox Z.D., Crimp J.L., Littleford H.E., Jowdry A.L., Jackman W.R. (2015). Hedgehog signaling regulates dental papilla formation and tooth size during zebrafish odontogenesis. Dev. Dyn..

[53] Javidan Y., Schilling T.F. (2004). Development of cartilage and bone. Methods Cell Biol..

[54] Guillot R., Muriach B., Rocha A., Rotllant J., Kelsh R.N., Cerdá-Reverter J.M. (2016). Thyroid hormones regulate zebrafish melanogenesis in a gender-specific manner. PLoS ONE.

[55] Hall B.K. (2008). The neural crest and neural crest cells: Discovery and significance for theories of embryonic organization. J. Biosci..

[56] Trainor P.A., Melton K.R., Manzanares M. (2003). Origins and plasticity of neural crest cells and their roles in jaw and craniofacial evolution. Int. J. Dev. Biol..

[57] Sant’Anna L., Tosello D. (2006). Fetal alcohol syndrome and developing craniofacial and dental structures—A review. Orthod. Craniofac. Res..

[58] Blanck-Lubarsch M., Dirksen D., Feldmann R., Sauerland C., Hohoff A. (2019). Tooth malformations, DMFT index, speech impairment and oral habits in patients with fetal alcohol syndrome. Int. J. Environ. Res. Public Health.

[59] Sltuckey E., Blerry C. (1984). The effects of high dose sporadic (binge) alcohol intake in mice. J. Pathol..

[60] Bloomquist R.F. (2008). Chemical Manipulation of Dental Patterning in Malawi Cichlids. http://hdl.handle.net/1853/21816.

[61] Sant’Anna L.B., Tosello D.O., Pasetto S. (2005). Effects of maternal ethanol intake on immunoexpression of epidermal growth factor in developing rat mandibular molar. Arch. Oral Biol..

[62] Matthiessen M., Rømert P. (1988). Changes of secretory ameloblasts in mini-pig fetuses exposed to ethanol in vivo. J. Dent. Res..

[63] Terry D.E., Chopra R.K., Ovenden J., Anastassiades T.P. (2000). Differential use of Alcian blue and toluidine blue dyes for the quantification and isolation of anionic glycoconjugates from cell cultures: Application to proteoglycans and a high-molecular-weight glycoprotein synthesized by articular chondrocytes. Anal. Biochem..

[64] Murawski N.J., Moore E.M., Thomas J.D., Riley E.P. (2015). Advances in Diagnosis and Treatment of Fetal Alcohol Spectrum Disorders: From Animal Models to Human Studies. Alcohol Res..

[65] Lovely C.B. (2020). Animal models of gene–alcohol interactions. Birth Defects Res..

[66] Becker H.C., Diaz-Granados J.L., Randall C.L. (1996). Teratogenic actions of ethanol in the mouse: A minireview. Pharmacol. Biochem. Behav..

[67] Raterman S.T., Metz J.R., Wagener F., Von den Hoff J.W. (2020). Zebrafish Models of Craniofacial Malformations: Interactions of Environmental Factors. Front. Cell Dev. Biol..

[68] Fushimi S., Wada N., Nohno T., Tomita M., Saijoh K., Sunami S., Katsuyama H. (2009). 17beta-Estradiol inhibits chondrogenesis in the skull development of zebrafish embryos. Aquat. Toxicol..

[69] Jiang Y.-J., Brand M., Heisenberg C.-P., Beuchle D., Furutani-Seiki M., Kelsh R.N., Warga R.M., Granato M., Haffter P., Hammerschmidt M. (1996). Mutations affecting neurogenesis and brain morphology in the zebrafish, Danio rerio. Development.

[70] Schilling T.F., Piotrowski T., Grandel H., Brand M., Heisenberg C.-P., Jiang Y.-J., Beuchle D., Hammerschmidt M., Kane D.A., Mullins M.C. (1996). Jaw and branchial arch mutants in zebrafish I: Branchial arches. Development.

[71] Halpern M.E., Ho R.K., Walker C., Kimmel C.B. (1993). Induction of muscle pioneers and floor plate is distinguished by the zebrafish no tail mutation. Cell.

[72] Odenthal J., Haffter P., Vogelsang E., Brand M., Van Eeden F., Furutani-Seiki M., Granato M., Hammerschmidt M., Heisenberg C.-P., Jiang Y.-J. (1996). Mutations affecting the formation of the notochord in the zebrafish, Danio rerio. Development.

